# The use of peripheral CD3^+^γδ^+^Vδ2^+^ T lymphocyte cells in combination with the ALBI score to predict immunotherapy response in patients with advanced hepatocellular carcinoma: a retrospective study

**DOI:** 10.1007/s00432-024-05896-y

**Published:** 2024-07-25

**Authors:** Shuhan Zhang, Luyang Li, Chengli Liu, Meng Pu, Yingbo Ma, Tao Zhang, Jiaqi Chai, Haoming Li, Jun Yang, Meishan Chen, Linghong Kong, Tian Xia

**Affiliations:** 1 Department of Hepatobiliary Surgery, Air Force Medical Center, PLA, Air Force Medical University, Beijing, China; 2 Postgraduate Training Base of Air Force Medical Center, China Medical University, Beijing, China; 3 Graduate School of Hebei North University, Zhangjiakou, China; 4 Department of Colorectal Surgery, 731 Hospital of China Aerospace Science and Industry group, Beijing, China; 5 Department of Ultrasound, Strategic Support Force Xingcheng Specialized Sanatorium, Huludao, China

**Keywords:** Hepatocellular carcinoma, Immunotherapy, T lymphocyte subsets, Γδ T cell

## Abstract

**Background:**

Currently, there is a lack of effective indicators for predicting the efficacy of immunotherapy in patients with advanced hepatocellular carcinoma (HCC). This study aimed to investigate the expression and prognostic value of peripheral T lymphocyte subsets in advanced HCC.

**Methods:**

Patients with advanced HCC who were treated with immune checkpoint inhibitors (ICIs) from December 2021 to December 2023 were included in the study. Flow cytometry was used to detect lymphocyte subsets before treatment. The patients were divided into disease control (DC) and nondisease control (nDC) groups based on treatment efficacy. Relationships between the clinical characteristics/peripheral T lymphocytes and immunotherapy efficacy were analyzed. The effectiveness of peripheral T lymphocyte subsets in predicting immunotherapy efficacy for patients with advanced HCC was analyzed using receiver operating characteristic (ROC) curves.

**Results:**

A total of 40 eligible patients were included in this study. Non-DC was significantly associated with higher albumin-bilirubin (ALBI) scores. The percentages of γδ^+^Vδ2^+^PD1^+^ T cells and γδ^+^Vδ2^+^Tim3^+^ T cells were greater in the nDC group than in the DC group. Multivariable regression analysis revealed that the ALBI score and T lymphocytes expressing γδ^+^Vδ2^+^PD1^+^ and γδ^+^Vδ2^+^Tim3^+^ were founded to be independent influencing factors. The area under the ROC curve (AUC) values for these combinations was 0.944 (95% CI, 0.882 ~ 1.000).

**Conclusions:**

The calculation of the ALBI score and determination of the percentages CD3^+^γδ^+^Vδ2^+^PD1^+^ T lymphocytes and CD3^+^γδ^+^Vδ2^+^Tim3^+^ T lymphocytes in the peripheral blood of patients with advanced HCC are helpful for predicting the patients’ responses to ICIs, helping to screen patients who may clinically benefit from immunotherapy.

**Retrospectively registered:**

number: ChiCTR2400080409, date of registration: 2024-01-29.

**Supplementary Information:**

The online version contains supplementary material available at 10.1007/s00432-024-05896-y.

## Introduction

With an increase of approximately 850,000 new cases every year, liver cancer is the third most prevalent malignancy and the sixth most common cause of cancer-related mortality (Sung et al. 2021). The prognosis of all hepatocellular carcinoma (HCC) stages is commonly evaluated using current models for liver cancer staging, such as the Barcelona Clinic Liver Cancer (BCLC) staging system. Curative treatment can be adopted for patients with early HCC, and approximately half of patients with HCC can be diagnosed early (Singal and El-Serag 2015). However, the prognosis for patients with advanced HCC is poor; the 5-year overall survival rate for these patients is only 14.1% (Allemani et al. 2018). Therefore, more effective treatment options must be developed.

Current research shows that the objective remission rate of patients with advanced hepatocellular carcinoma (HCC) treated with immune checkpoint inhibitors (ICIs) in combination with molecular targeted agents is approximately 30% (Finn et al. 2020; Ren et al. 2021; Kelley et al. 2022). Therefore, it is clinically important to identify the subgroups that respond well to ICIs before initial treatment. At present, in the treatment of advanced HCC, there have been few studies related to the status of peripheral blood immune function as a biological marker for the efficacy of ICIs. Current research shows that increased circulating CD8^+^ T lymphocytes in patients with HCC receiving pembrolizumab can result in improved therapeutic effects (Hong et al. 2022). The lower percentage of baseline granulocytes expressing programmed death-1 (PD-1) in patients with HCC receiving atezolizumab-Bevacizumab indicates that these drugs may have better therapeutic effects (Giovannini et al. 2023). Therefore, we speculate that the function and depletion of immune cell subsets may be powerful tools for predicting immunotherapy efficacy.

In this study, we explored the molecular and immunologic characteristics of peripheral γδ^+^ T lymphocyte subsets and their combined use with the albumin-bilirubin (ALBI) score to predict immunotherapy efficacy for patients with advanced HCC.

## Methods

### Study population selection

This study was approved by the Medical Ethics Committee of the Air Force Medical Center (No. 2021-175-PJ01) and registered with the China Clinical Trials Registry under registration number ChiCTR2400080409. Patients who were diagnosed with advanced HCC at the Air Force Medical Center between December 2021 and December 2023 were included in this study. The inclusion criteria were as follows: (1) the patient was diagnosed with primary HCC by hepatic histological examination or clinical evidence; (2) there was at least one measurable substantial lesion in the liver; (3) the Child-Pugh liver function classification was A or B (score ≤ 7); (4) the BCLC classification was stage B or C; and (5) the patient voluntarily accepted the combination therapy of camrelizumab and lenvatinib and completed at least 6 cycles of treatment. The exclusion criteria were as follows: (1) patients complicated with other malignant tumors; (2) patients with dysfunction of the heart, brain, lung, kidney or other vital organs; (3) patients with severe coagulation disorders, bleeding tendency, or receiving anticoagulant and thrombolytic therapy; (4) patients with hypertension that was difficult to control with medication; (5) patients with a history of immunodeficiency disease, previous organ transplantation, or severe bone marrow suppression; and (6) patients with definite contraindications to the drugs used in this study.

### Treatment plan and follow-up

The specific plan was to administer lenvatinib (Weicai China Pharmaceutical Company, Import Registration Certificate No. H20180052) orally once a day (body weight < 60 kg: 8 mg; body weight ≥ 60 kg: 12 mg) and to receive intravenous infusions of camrelizumab (Suzhou Shengdiya Biopharmaceutical Co., Ltd, National Drug Registration Number: S20190027, 200 mg) every two weeks. Enhanced MRI or CT was performed every 3 cycles (6 weeks) to evaluate efficacy. Outcome, including complete response (CR), partial response (PR), disease progression (PD), disease stability (SD) were evaluated according to the modified solid tumor clinical efficacy evaluation criteria (MRECIST) version 1.1. The disease control (DC) group was defined as the CR + PR + SD group. Patients with PD and deceased patients were included in the nondisease control (nDC) group.

### Data collection

The laboratory testing of peripheral blood was performed by the Department of Clinical Laboratory. The immune function indices of peripheral T lymphocyte subsets were measured using a flow cytometry system. Before treatment, all study subjects fasted and approximately 3 ml of peripheral venous blood was collected in a tube. Erythrocytes were lysed using RBC lysis buffer for 10 min in the dark. Three milliliters of phosphate-buffered saline (PBS) was added to wash the cells. After centrifugation at 1500 r/min for 5 min, an additional 1 mL of PBS was added for further mixing. For intracellular cytokine expression, cells were stimulated with phorbol myristate acetate (PMA, Sigma-Aldrich, Germany) and brefeldin A (BFA, BD Biosciences, USA) at 37 ºC for 5 h. The following monoclonal antibodies were used: APC-H7 Mouse Anti-Human CD3 (BD Bioscience, USA); BV605 Mouse Anti-Human CD4 (BD Bioscience, USA); FITC Mouse Anti-Mouse CD8 (BD Bioscience, USA); BV421 Mouse Anti-Human γδ TCR (BD Bioscience, USA); PE Mouse Anti-Human CD279 (BD Bioscience, USA); APC Anti-Human CD366 (Tim-3, BioLegend, USA); Brilliant Violet 510 Anti-Human TCR Vδ2 (BioLegend, USA); PerCP-Vio 700 Anti-Human TCR Vδ1 (Miltenyi Biotec, Germany); and PE-Cy7 Mouse Anti-Human CD28 (BD Bioscience, USA). These antibodies were added to the bottom of the flow tube and mixed thoroughly. The stained cells were analyzed by flow cytometry using a FACS Calibur cytometer equipped with CellQuest software.

The following data were collected and recorded: (I) baseline characteristics, including age, sex, diabetes status, hypertension status, hepatitis B virus (HBV) status, hepatitis C virus (HCV) status, portal vein tumor thrombus (PVTH) status, American Joint Committee on Cancer (AJCC) TNM stage, and BCLC stage; (II) various evaluation indicators, including the ALBI score, neutrophil-to-lymphocyte ratio (NLR), platelet-to-lymphocyte ratio (PLR), α-fetoprotein (AFP) level and lactate dehydrogenase (LDH) level; and (III) the functional status of T lymphocyte subsets, decline in T lymphocyte subsets and T-lymphocyte immune checkpoint analysis.

### Statistical analysis

Continuous data with a normal distribution were compared using t tests and are presented as the mean ± standard deviation (SD). Continuous data with skewed distributions were compared using the Mann-Whitney U test and are presented as interquartile ranges (IQRs). Categorical data were analyzed by the chi-squared test or Fisher’s exact test. Univariable and multivariable logistic regressions were used to analyze potential independent risk factors for the efficacy of immunotherapy. Receiver operating characteristic (ROC) curves were used to analyze the efficacy of related factors in predicting immunotherapy efficacy for advanced HCC.

## Results

### Demographic and clinical characteristics

**Fig. 1:**
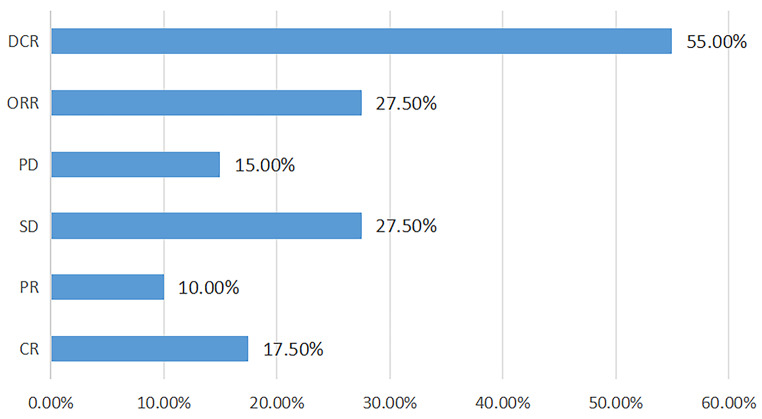
Therapeutic outcomes of patients. DCR: disease control rate, ORR: objective response rate, PD: disease progression, SD: disease stability, PR: partial response, CR: complete response

A total of 40 enrolled patients with advanced HCC (23 males, 17 females; mean age, 55.9 years) were enrolled and divided into a DC group (*n* = 22) and a nDC group (*n* = 18). The median follow-up time of patients was 16 months. The median survival time of patients was 22 months. Figure 1 presents detailed therapeutic outcomes. Table 1 presents the clinical and demographic characteristics of the study cohort. No significant differences were found in sex, age, diabetes status, hypertension status, HBV status, HCV status, PVTH status, BCLC stage, AJCC stage, T stage, N stage, M stage, AFP level, cirrhosis status, LDH level, NLR or PLR between the two groups. The ALBI score was greater in the nDC group (*p* < 0.05). Online Resourse 1 Supplementary Material 1 presents pathological and immunohistochemical data of several patients.

**Table 1:** Clinical and demographic features of eligible patients

Variables	Total (*n* = 40)	DC (*n* = 22)	nDC (*n* = 18)	*P*-value
Gender				0.676
Male	23 (57.5%)	12 (54.5%)	11 (61.1%)	
Female	17 (42.5%)	10 (45.5%)	7 (38.9%)	
Age, years	55.9 ± 13.4	52.5 ± 11.6	60.1 ± 14.6	0.072
Diabetes				0.613
No	36 (90.0%)	19 (86.4%)	17 (94.4%)	
Yes	4 (10.0%)	3 (13.6%)	1 (5.6%)	
Hypertension				0.564
No	27 (67.5%)	14 (63.6%)	13 (72.2%)	
Yes	13 (32.5%)	8 (36.4%)	5 (27.8%)	
HBV				0.999
No	30 (75.0%)	16 (72.7%)	14 (77.8%)	
Yes	10 (25.0%)	6 (27.3%)	4 (22.2%)	
HCV				0.149
No	31 (77.5%)	15 (68.2%)	16 (88.9%)	
Yes	9 (22.5%)	7 (31.8%)	2 (11.1%)	
PVTH				0.68
No	33 (82.5%)	19 (86.4%)	14 (77.8%)	
Yes	7 (17.5%)	3 (13.6%)	4 (22.2%)	
BCLC stage				0.899
1	22 (55.0%)	13 (59.1%)	9 (50%)	
2	9 (22.5%)	5 (22.7%)	4 (22.2%)	
3	9 (22.5%)	4 (18.2%)	5 (27.8%)	
AJCC stage				0.504
II	13 (32.5%)	9 (40.9%)	4 (22.2%)	
III	18 (45.0%)	9 (40.9%)	9 (50%)	
IV	9 (22.5%)	4 (18.2%)	5 (27.8%)	
T stage				0.458
T2	13 (32.5%)	9 (40.9%)	4 (22.2%)	
T3	20 (50.0%)	10 (45.5%)	10 (55.6%)	
T4	7 (17.5%)	3 (13.6%)	4 (22.2%)	
N stage				1
N0	36 (90.0%)	20 (90.9%)	16 (88.9%)	
N1	4 (10.0%)	2 (9.1%)	2 (11.1%)	
M stage				0.579
M0	37 (92.5%)	21 (95.5%)	16 (88.9%)	
M1	3 (7.5%)	1 (4.5%)	2 (11.1%)	
Cirrhosis				0.404
No	20 (50%)	10 (45.5%)	10 (55.6%)	
Yes	20 (50%)	12 (54.5%)	8 (44.4%)	
AFP, ng/ml	246.9 ± 446.4	361.4 ± 493.4	107.0 ± 344.6	0.072
LDH, ng/ml	333.0 ± 212.8	280.2 ± 138.2	397.5 ± 268.8	0.083
NLR	3.3 ± 2.7	3.0 ± 2.5	3.8 ± 3.0	0.362
PLR	129.2 ± 85.0	123.0 ± 81.8	136.9 ± 90.5	0.612
ALBI	-2.4 ± 0.6	-2.6 ± 0.4	-2.2 ± 0.7	0.014

DC: disease control; HBV: hepatitis B virus; HCV: hepatitis C virus; PVTH: portal vein tumor thrombus; BCLC: Barcelona Clinic Liver Cancer; AJCC: American Joint Committee on Cancer; AFP: α-fetoprotein; LDH: lactate dehydrogenase; NLR: neutrophil to lymphocyte ratio, PLR: platelet to lymphocyte ratio, ALBI: albumin bilirubin

#### Online Resource 1

Pathological and immunohistochemistry results of patients.

### Peripheral T lymphocyte subsets

**Fig. 2:**
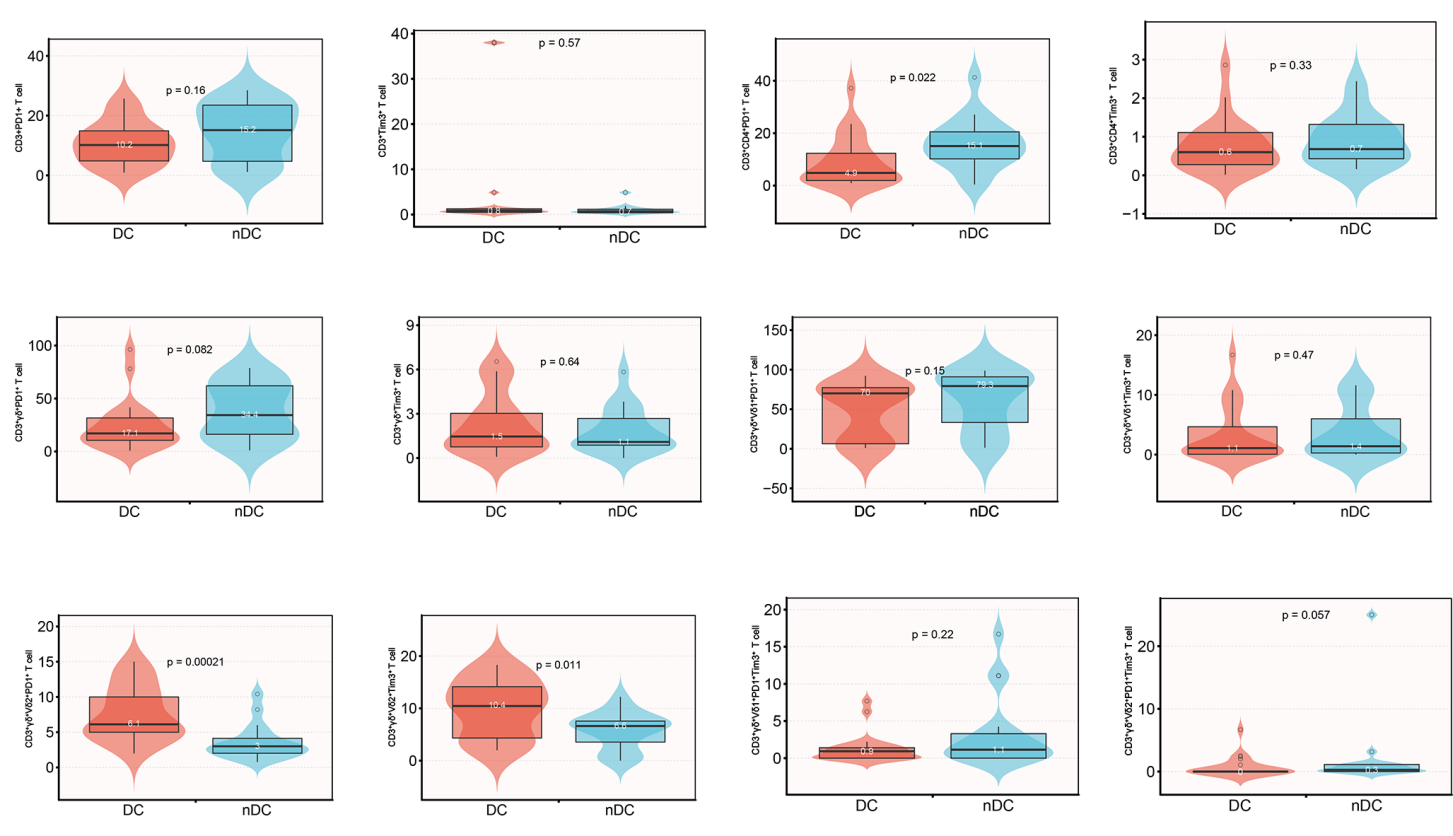
Distribution of T lymphocyte subsets in DC and nDC group

Table 2 shows the levels of T lymphocyte subsets in the DC and nDC groups. The percentage of CD3^+^γδ^+^Vδ1^+^ T cells was greater in the active TED group than in the inactive TED group (*P* < 0.05), whereas no significant differences were detected in other lymphocyte subsets between the two groups (all *P* > 0.05). Figure 2 shows the analysis of immune checkpoints in peripheral T lymphocyte subsets. The percentages of CD3^+^γδ^+^Vδ2^+^ PD1^+^ T cells and CD3^+^γδ^+^Vδ2^+^ Tim3^+^ T cells were elevated in the DC group. Online Resource 2 shows the raw data of flow cytometry.

**Table 2:** Peripheral lymphocyte subsets in patients with advanced HCC.

Variables	Total (*n* = 40)	DC (*n* = 22)	nDC (*n* = 18)	*P*-value
CD3^+^CD4^+^T cell (%)	52.5 ± 17.0	54.6 ± 18.4	49.9 ± 15.1	0.394
CD3^+^CD8^+^T cell (%)	43.5 ± 21.4	45.0 ± 26.4	41.5 ± 13.3	0.612
TH/TC cell (%)	1.5 ± 0.8	1.6 ± 0.9	1.4 ± 0.8	0.651
CD3^+^γδ^+^ T cell (%)	4.5 ± 4.2	4.2 ± 3.4	4.9 ± 5.0	0.580
CD3^+^γδ^+^Vδ1^+^T cell (%)	29.5 ± 27.2	21.5 ± 17.6	39.3 ± 33.5	0.037
CD3^+^γδ^+^Vδ2^+^T cell (%)	26.1 ± 23.2	27.5 ± 21.9	24.4 ± 25.2	0.671
Vδ1^+^/Vδ2^+^T cell (%)	4.0 ± 10.7	1.6 ± 2.3	6.8 ± 15.5	0.134
CD3^+^γδ^+^CD28^+^T cell (%)	45.5 ± 23.1	47.7 ± 19.0	42.8 ± 27.6	0.510
CD3^+^γδ^+^Vδ1^+^CD28^+^T cell (%)	30.2 ± 27.1	30.7 ± 25.7	29.6 ± 29.4	0.897
CD3^+^γδ^+^Vδ2^+^CD28^+^T cell (%)	77.9 ± 23.1	74.7 ± 26.1	81.7 ± 18.7	0.345

DC: disease control

#### Online Resource 2

The raw data of flow cytometry.

### Logistic analysis for patients with advanced HCC

**Fig. 3:**
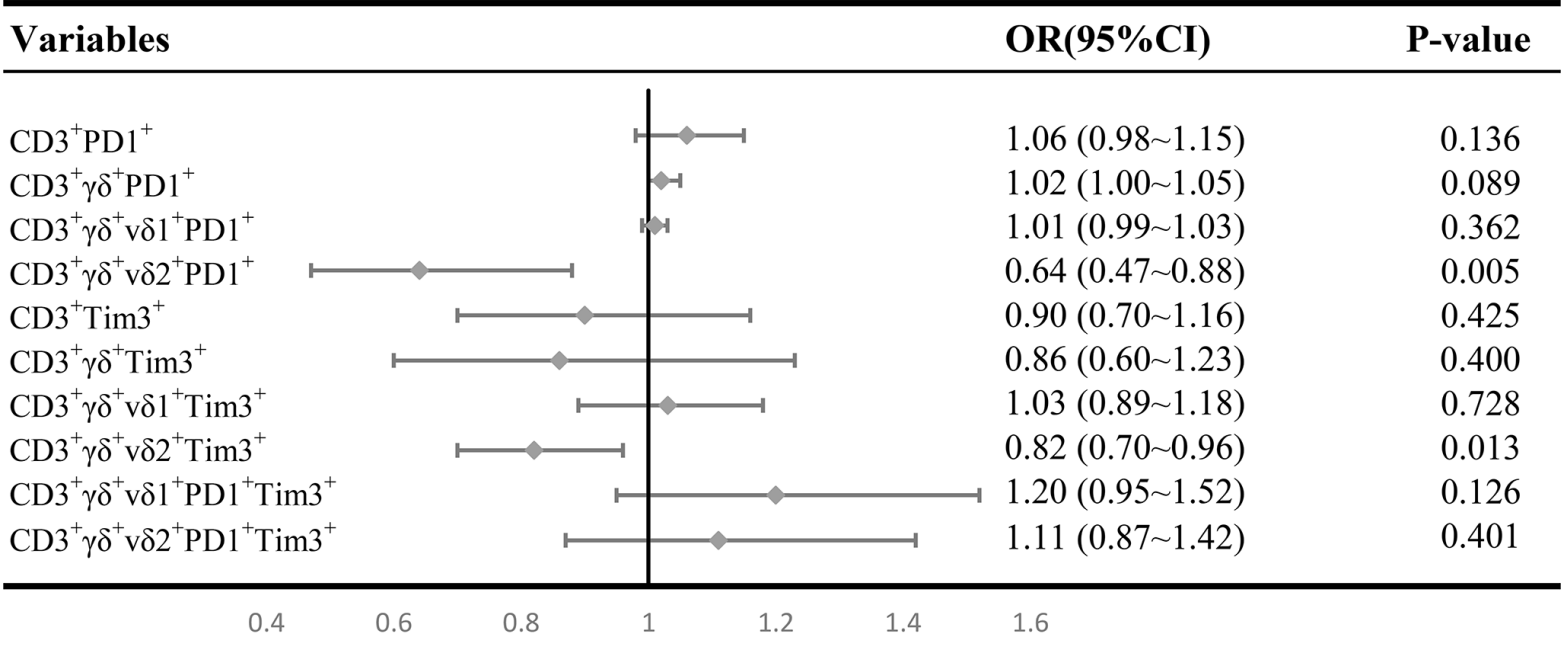
Forestplot of the efficacy of ICIs were analyzed by the multivariable analysis

Logistic regression analyses were performed to identify the factors related to immunotherapy efficacy. Univariable logistic regression analysis revealed that the ALBI score was a risk factor (OR: 5.35, *p* < 0.05), while CD3^+^γδ^+^Vδ2^+^PD1^+^ T cells (OR: 0.64, *p* < 0.05) and CD3^+^γδ^+^Vδ2^+^Tim3^+^ T cells (OR: 0.82, *p* < 0.05) were protective factors (Table 3; Fig. 3). These factors were further included in the multivariable regression analysis, and the same results were the same (Table 4).

**Table 3:** Univariable logistics analysis in patients with advanced hepatocellular carcinoma

Variables	OR (95%CI)	*P* value
Gender		
Male	Reference	
Female	0.76 (0.22 ~ 2.71)	0.676
Age	1.05 (0.99 ~ 1.10)	0.079
Diabetes		
No	Reference	
Yes	0.37 (0.04 ~ 3.93)	0.411
Hypertension		
No	Reference	
Yes	0.67 (0.17 ~ 2.59)	0.565
HBV		
No	Reference	
Yes	0.76 (0.18 ~ 3.26)	0.714
HCV		
No	Reference	
Yes	1.02 (1.00 ~ 1.05)	0.134
PVTH		
No	Reference	
Yes	1.81 (0.35 ~ 9.41)	0.481
BCLC		
1	Reference	
2	1.16 (0.24 ~ 5.53)	0.856
3	1.81 (0.38 ~ 8.64)	0.459
AJCC stage		
II	Reference	
III	2.25 (0.5 ~ 10.05)	0.288
IV	2.81 (0.48 ~ 16.43)	0.251
T stage		
T2	Reference	
T3	2.25 (0.52 ~ 9.77)	0.279
T4	3 (0.45 ~ 20.15)	0.258
N stage		
N0	Reference	
N1	1.25 (0.16 ~ 9.88)	0.832
M stage		
M0	Reference	
M1	2.62 (0.22 ~ 31.57)	0.447
Cirrhosis		
No	Reference	
Yes	0.67 (0.19 ~ 2.33)	0.526
AFP	1.00 (1.00 ~ 1.00)	0.108
LDH	1.00 (1.00 ~ 1.01)	0.099
NLR	1.12 (0.88 ~ 1.43)	0.362
PLR	1.00 (0.99 ~ 1.01)	0.603
ALBI	5.35 (1.19 ~ 24.01)	0.028

HBV: hepatitis B virus; HCV: hepatitis C virus; PVTH: portal vein tumor thrombus; BCLC: Barcelona Clinic Liver Cancer; AJCC: American Joint Committee on Cancer; AFP: α-fetoprotein; LDH: lactate dehydrogenase; NLR: neutrophil to lymphocyte ratio, PLR: platelet to lymphocyte ratio, ALBI: albumin bilirubin

**Table 4:** Multivariable logistics analysis in patients with advanced hepatocellular carcinoma

Variables	OR	95%CI	*P*-value
ALBI	48.12	2.68 ~ 863.51	0.009
CD3^+^γδ^+^vδ2^+^PD1^+^T cell	0.50	0.31 ~ 0.82	0.006
CD3^+^γδ^+^vδ2^+^Tim3^+^T cell	0.68	0.47 ~ 0.99	0.042

ALBI: albumin bilirubin

### Construction of prediction models

**Fig. 4:**
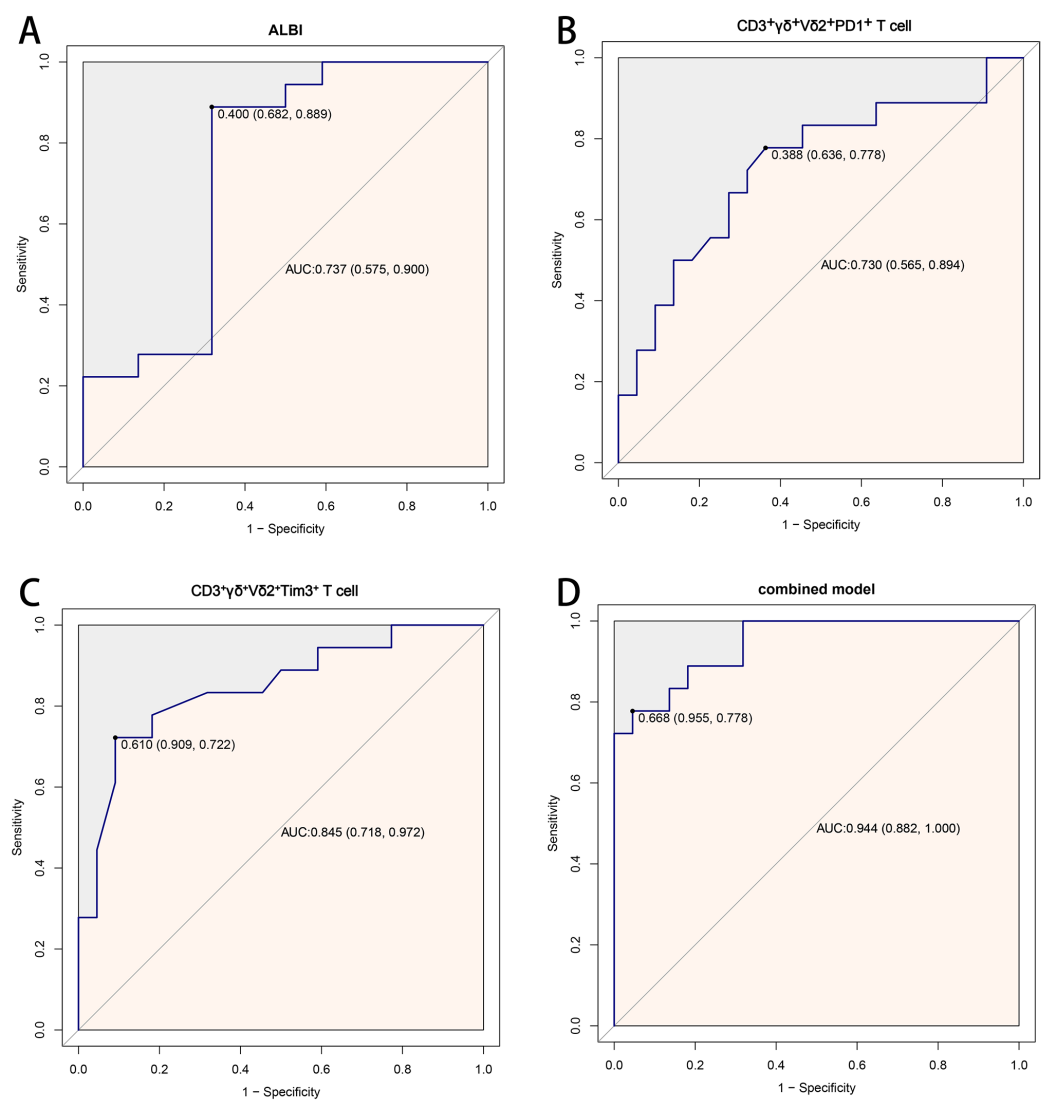
The ROC curve graph of T lymphocyte subsets and ALBI in patients with advanced HCC. **A**, ALBI ROC curve; **B**, CD3^+^γδ^+^Vδ2^+^PD1^+^ T cell ROC curve; **C**, CD3^+^γδ^+^Vδ2^+^Tim3^+^ T cell ROC curve; **D**, combined model ROC curve. ROC: receiver operating characteristic; ALBI: albumin-bilirubin

The ROC curve results are shown in Fig. 4. The AUCs of the ALBI score, CD3^+^γδ^+^Vδ2^+^PD1^+^ T-cell and CD3^+^γδ^+^Vδ2^+^Tim3^+^ T-cell counts were 0.737 (95% CI: 0.575 ~ 0.900), 0.730 (95% CI: 0.565 ~ 0.894), and 0.845 (95% CI: 0.718 ~ 0.972), respectively. The combined model showed an AUC of 0.944 (95% CI, 0.882 ~ 1.000) for all patients. These results suggest that the predictive ability of the combined score is superior to those of the other three indicators.

## Discussion

In this study, we analyzed the differences in the numbers of T lymphocyte subsets in peripheral blood samples collected before immunotherapy. We concluded that the ALBI score, γδ^+^Vδ2^+^PD1^+^ T cells (%), and γδ^+^Vδ2^+^Tim3^+^ T cells (%) were significantly correlated with immunotherapy efficacy for patients with advanced HCC. Thus, the combined model can predict immunotherapy efficacy well.

γδ T cells account for approximately 1–5% of the total number of T cells in the peripheral blood, but their proportion is greater in epithelial tissues where tumors occur. Many studies have shown that γδT cells can function as cytotoxic cells to participate in the antitumor immune response (Liu et al. 2022; Silva-Santos and Mensurado 2023). γδ T cells represent a unique subset of lymphocytes that exhibit both innate and acquired immunity. γδ T cells adopt a different T-cell receptor (TCR) structure composed of a γ chain and a δ chain. According to differences in their δ chains, human γδT cells are mainly divided into three subsets, namely, Vδ1, Vδ2 and Vδ3 T cells. Vδ2^+^ γδ T cells are mainly found in the peripheral blood, and γδ T cells expressing Vγ9^+^Vδ2^+^ TCRs account for 50–95% of the total number of γδ T cells, which are the cells that mainly exert antitumor effects (Yin et al. 2024). Γδ T cells can directly recognize tumor-associated antigens in a nonlimiting manner through the major histocompatibility complex (MHC), kill a variety of tumor cells, and secrete a variety of antitumor cytokines to enhance the antitumor immune response (Willcox et al. 2020; Park et al. 2021). Therefore, γδ T cells play an important role in the immune system’s response to cancer and crucial positive roles in the responses of the innate and adaptive immune systems to cancer (Ni et al. 2020; Kabelitz et al. 2020; Lee et al. 2022).

The tumor microenvironment of liver cancer consists of a complex environment composed of cancer cells, cytokines, immune cells, nontumor cells and the extracellular matrix. These components interact with each other, producing various cytokines, chemokines and other factors involved in immune suppression and immune escape. PD-1 and Tim3 are immune checkpoints on the surface of T cells. Recent studies have shown that high expression levels of PD-1 and Tim3 can induce peripheral immune tolerance and inhibit antitumor immunity (Zhang et al. 2024; Abdelrahman et al. 2024). In this study, the percentages of Vδ2^+^PD1^+^ and Vδ2^+^Tim3^+^ T cells in the DC group were greater than those in the control group, suggesting that the Vδ2^+^ T-cell antitumor immune response was inhibited in these patients. According to the research of Lawrence Fong et al., γδ^+^Vδ2^+^ T cells infiltrated by renal cell carcinoma can simultaneously express molecular markers such as PD-1, TIGIT, and Tim3, indicating that the antitumor immune response is inhibited, while such cells are not observed in normal tissues. However, γδ^+^Vδ2^+^ T cells can still secrete cytokines and perforin to kill tumor cells, which is related to the clinical benefits of improving the immune microenvironment after ICI therapy (Rancan et al. 2023). Tumor immunotherapy is a major breakthrough in the field of cancer treatment. Since 2017, immune checkpoint inhibitors, such as nivolumab, pembrolizumab, camrelizumab and atezolizumab, which are PD-1/PDL-1 and CTLA-4 inhibitors, have been widely applied to treat hepatocellular carcinoma. However, the response rate to single-drug immunotherapy is low; for example, the objective response rate (ORR) of nivolumab is 20%, and the ORR of pembrolizumab is only 17%, which may be due to the complexity of the tumor immune microenvironment and the interactions of various immune molecules. In our study, patients with high PD1^+^ and Tim3^+^ levels in Vδ2^+^ T cells were more likely to benefit from PD1 treatment, while recent studies have shown that butyrophilin-like protein (BTN) plays a key role in the activation of Vγ9^+^Vδ2^+^ T cells and the recognition of tumor cells (Du et al. 2022; Lee et al. 2022; Hu et al. 2023). Further study on this mechanism by Alexander Marson et al. in 2023 revealed that tumor cells lacked regulatory metabolic genes, and excessive cholesterol was produced. These factors led to an increase in and activation of BTN expression on the cell surface, which was more easily recognized by γδ T cells and enhanced Vγ9^+^Vδ2^+^ T-cell receptor-mediated tumor killing (Mamedov et al. 2023). This indicates that the use of immune checkpoint inhibitors to reverse the immunosuppressive state of γδ^+^Vδ2^+^ T cells plays an important role in tumor immunotherapy.

The ALBI is a scoring method developed by Johnson et al. in 2015 to provide a new assessment of liver function in patients with HCC (Johnson et al. 2015). Unlike the classic Child-Pugh score, the ALBI score eliminates two subjective scoring indicators, ascites and hepatic encephalopathy, providing a faster and more objective methods to assess liver function in clinical practice. Previous studies have shown that patients with high ALBI scores have a worse prognosis than those with low ALBI scores (Yoshino et al. 2024; Tian et al. 2024; Wonglhow et al. 2024). This may be because patients with HCC with better liver function have a stronger immune response to tumor production during liver resistance, which in turn inhibits tumor growth and invasion, thereby delaying recurrence. Our results also support previous research. In this study, we also included several indicators from peripheral blood, such as NLR and PLR. Previous research concluded that high NLR and PLR are also associated with poor prognosis in patients with HCC (Sangro et al. 2020; Liu et al. 2024; Öcal et al. 2024). However, in this study, we failed to observe a correlation between NLR or PLR and tumor response, which may be due to the relatively small number of enrolled patients.

Our study also has several limitations. First, the sample size of this study was relatively small. The expression of various subsets of γδ T cells should be further verified by various methods. Second, research on the effect of γδ T cells on the depletion of functional T cell is insufficient. Subsequently, sequencing of clinical samples and mouse tumor model experiments will be conducted to explore their effects in vivo and in vitro and to analyze the potential upstream and downstream molecules and related signaling pathways of various subsets of γδ T cells.

## Conclusions

In summary, the calculation of the ALBI score and determination of peripheral CD3^+^γδ^+^Vδ2^+^PD1^+^ T lymphocyte cells and CD3^+^γδ^+^Vδ2^+^Tim3^+^ T lymphocyte cells in patients with advanced HCC are conducive to predicting the efficacy of ICIs and screening patients who may benefit from immunotherapy clinically.

### Electronic supplementary material

Below is the link to the electronic supplementary material.

Supplementary Material 1

Supplementary Material 2

## Data Availability

No datasets were generated or analysed during the current study.
